# Bipolar Cu/HfO_2_/p^++^ Si Memristors by Sol-Gel Spin Coating Method and Their Application to Environmental Sensing

**DOI:** 10.1038/s41598-019-46443-x

**Published:** 2019-07-10

**Authors:** Sabina Abdul Hadi, Khaled M. Humood, Maguy Abi Jaoude, Heba Abunahla, Hamda Faisal Al Shehhi, Baker Mohammad

**Affiliations:** 1 Department of Electrical and Computer Engineering, Khalifa University of Science and Technology, P.O. Box 127788, Abu Dhabi, UAE; 2 Currently working at College of Engineering and IT, University of Dubai, P.O. Box 14143, Dubai, UAE; 3 Department of Chemistry, Khalifa University of Science and Technology, P.O. Box 127788, Abu Dhabi, UAE; 4 UAE Space Agency, 7133 Abu Dhabi, UAE

**Keywords:** Electrical and electronic engineering, Electronic devices

## Abstract

In this paper, the memristive switching behavior of Cu/ HfO_2_/p^++^ Si devices fabricated by an organic-polymer-assisted sol-gel spin-coating method, coupled with post-annealing and shadow-mask metal sputtering steps, is examined. HfO_2_ layers of about 190 nm and 80 nm, are established using cost-effective spin-coating method, at deposition speeds of 2000 and 4000 rotations per minute (RPM), respectively. For two types of devices, the memristive characteristics (*V*_*on*_, *I*_*on*_, and *V*_*reset*_) and device-to-device electrical repeatability are primarily discussed in correlation with the oxide layer uniformity and thickness. The devices presented in this work exhibit an electroforming free and bipolar memory-resistive switching behavior that is typical of an Electrochemical Metallization (ECM) I-V fingerprint. The sample devices deposited at 4000 RPM generally show less variation in electrical performance parameters compared to those prepared at halved spin-coating speed. Typically, the samples prepared at 4000 RPM (n = 8) display a mean switching voltage *V*_*on*_ of 3.0 V (±0.3) and mean reset voltage *V*_*reset*_ of −1.1 V (±0.5) over 50 consecutive sweep cycles. These devices exhibit a large *R*_*off*_/*R*_*on*_ window (up to 10^4^), and sufficient electrical endurance and retention properties to be further examined for radiation sensing. As they exhibit less statistical uncertainty compared to the samples fabricated at 2000 RPM, the devices prepared at 4000 RPM are tested for the detection of soft gamma rays (emitted from low-activity Cs-137 and Am-241 radioactive sources), by assessing the variation in the on-state resistance value upon exposure. The analysis of the probability distributions of the logarithmic *R*_on_ values measured over repeated ON-OFF cycles, before, during and after exposing the devices to radiation, demonstrate a statistical difference. These results pave the way for the fabrication and development of cost-effective soft-gamma ray detectors.

## Introduction

The resistance switching capability of dielectric layers has been observed experimentally almost 50 years ago^1–3^. For memristors (MRs), or resistive random-access memory (ReRAM) devices, switching occurs between a high resistive state (HRS) and low resistive state (LRS) when an external stimulus, such as an applied electric field, results in the formation (and rupture) of conductive filaments (CFs) inside the dielectric film. Owing to a number of technological benefits such as high scalability, low power consumption and relatively fast read/write times^4^, memristive devices with different electrical properties and physical dimensions are being extensively explored for various applications including computing, hardware security^5–7^ and environmental sensing^7–12^.

Among a plethora of different dielectric materials exhibiting resistive switching behavior^13–15^, HfO_2_ has received much interest from the scientific community owing to its compatibility with the CMOS technology, low operating voltage, high dielectric constant and high thermodynamic stability^16–18^. Nano-scale HfO_2_ based memristors can exhibit either bipolar^17–21^ or unipolar^22–25^ switching, as well as both behaviors^26^. Their electrical behavior is largely influenced by the choice of top and bottom electrodes (TE and BE respectively), the presence of oxygen vacancy-rich interfacial layers, or the quality of the metal-oxide layer. Various techniques can be used to deposit HfO_2_ layers, including the atomic layer deposition (ALD)^19,25,27–29^, pulsed laser deposition^30^, metal organic and chemical vapor deposition (MOCVD or CVD)^31^, physical deposition by magnetron sputtering^20,32–34^ and more recently, sol-gel spin-coating method^35–44^. The ALD, CVD and sputtering techniques all provide a relatively good control over the deposited material properties and high yield. Nonetheless, they require the use of expensive specialized tools in cleanroom environment and are mainly suitable for the deposition of few nanometers thick films. On the other hand, the sol-gel spin coating route, due to its simplicity and cost-effectiveness, is being progressively explored for large-area device fabrication^45^, including thin film transistors (TFT)^46,47^ and nonvolatile resistive memories^35–44^.

**Figure 1:**
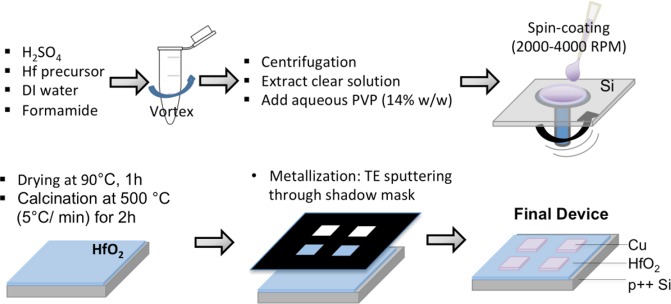
Process flow for preparation of Cu/HfO_2_/p^++^ Si spin-coated devices.

**Figure 2:**
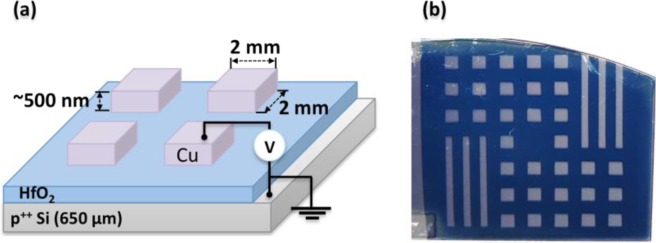
(**a**) Side view schematics of a sample, where each Cu TE square (2 mm × 2 mm) defines one memristor device and **(b)** top view image of one of the fabricated samples with many devices.

Here, we report on the preparation of 2 mm by 2 mm Cu-(TE)/HfO_2_/p^++^ Si-(BE) memristor devices via a sol-gel spin coating method, which involves the deposition of a composite polyvinylpyrrolidone (PVP) – HfO_2_ precursor thin films on a p^++^ Si substrate. The polymer controls the viscosity of the deposition mixture during the spin coating and additionally binds with the metal-oxide precursor species, to give a composite with a relatively uniform distribution of metal ions. The synthesis is coupled with a post-thermal annealing step to remove the organic polymer template, and a subsequent shadow mask sputtering step to deposit Cu TEs. The process flow for the preparation of the Cu/HfO_2_/p^++^ Si is summarized in Fig. 1. An illustration of the final device structure with approximate layers thicknesses and the top view optical image of a fabricated sample are all shown in Fig. 2.

In this study, the effects of the spin coating speed on the metal-oxide dielectric layer thickness and topography, as well as the corresponding current-voltage (I-V) characteristics and device-to-device performance are explored. The structure yielding less variability in the electrical performance parameters is further explored in radiation sensing by examining the changes in the electrical response upon exposure to low activity gamma ray sources. The statistical significance of the differences observed in the log(*R*_on_) values is evaluated as a qualitative performance indicator of the gamma ray transducing capability of these memristive devices.

## Results

### Morphology and thickness of the oxide layer

**Figure 3:**
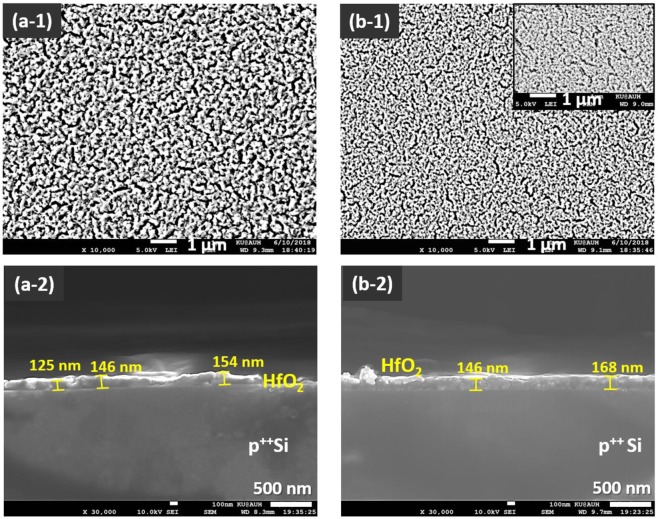
SEM images of the top views (**a-1**,**b-1**) and cross-section views (**a-2**,**b-2**) of HfO_2_/p^++^ Si regions from wafer samples A1 and A2, successively spin coated at a speed of 2000 RPM, using the same composite precursor mixture at room temperature. **(a-1**,**a-2)** sample A1; **(b-1**,**b-2)** sample A2. The inset in b-1 displays another top view area of sample A2. The results show a denser surface texture of the oxide layer in sample A2 (**b-1**), implying a solution-aging factor.

To examine the effect of sample preparation and aging on the batch-to-batch repeatability of the spun-coated layer morphology, two wafer samples (A1 and A2) were prepared at 2000 RPM, using the same deposition mixture. Aqueous PVP polymer, acting as a stress-relaxing and casting agent, was introduced in the mixture to enhance the adhesion of the metal-oxide precursors onto the wafers and obtain a metal-organic composite with suitable viscosity for uniform spin coating deposition^48–50^. The top-view SEM images of the coatings annealed at 500 °C (Fig. 3a-1,b-1) show uniformly rugged surfaces, consisting of interconnected HfO_2_ particles and a macroporous network.

The porosity, which is not observed in the as-prepared organic composite films (data not shown), can be ascribed to voids arising from thermally-induced stress factors. The latter include shear and compressive forces that develop during the post-drying/thermal annealing process, which involves loss of solvents, decomposition of the organic polymer content and subsequent sintering of the metal oxide grains^36,48^. Doi *et al*.^48^ have shown that the PVP concentration in the polymer-assisted spin coating deposition of metal oxides can greatly impact the formation of cracks and pores in the final film. Typically, too little of PVP cannot prevent the macroscopic cracks that are induced by the thermal shrinkage which is caused by the densification of the metal oxide network, whilst high polymer contents, may induce a porogenic templating effect, by generating voids through liquid expulsion via phase separation^49^. In comparison to sample A1, the denser surface texture of the oxide layer in sample A2 (Fig. 3a-1,b-1) infers a solution-aging factor. This can be explained by a readily enhanced condensation state of the metal alkoxides in the source mixture leading to an accelerated onset of the gelation step relative to phase separation. According to the cross-section views in Fig. 3(a-2,b-2), the average oxide film thickness is within a range of ~140 (±30 nm) for both samples A1 and A2. The larger variation in the thickness of the film across sample A1 is due to greater surface corrugations.

**Figure 4:**
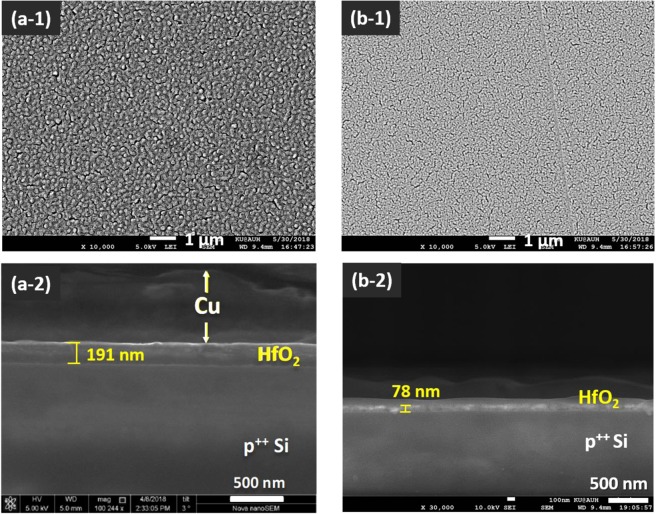
SEM images for top views (**a-1**,**b-1**) and cross section views (**a-2**, **b-2**) of calcined HfO_2_/p^++^ Si regions from samples produced at different spinning speeds. **(a-1**,**a-2)** sample A, 2000 RPM; **(b-1**,**b-2)** sample B, 4000 RPM. Sample A HfO_2_ has larger particle size and about 2.5-fold higher thickness compared to sample B.

In order to examine the impact of the spin coating speed on the thickness and uniformity of the dielectric HfO_2_ films, two different wafer samples, A and B were prepared at 2000 and 4000 RPM, respectively, as these speeds provided the best sample coverage for the used sol-gel solution viscosity. As noticed from the top-view SEM image of the calcined samples shown in Fig. 4(a-1,b-1), a relatively dense surface structure is equally obtained, yet with larger particle size formed at the lower spinning speed. The cross-sectional analyses of devices cut from the two wafers (Fig. 4(a-2,b-2)) show approximately a 2.5-fold thinner oxide layer formed when the spinning speed is doubled from 2000 to 4000 RPM. This observation agrees with the empirical trend documented for the change in the coating thickness of spun-coated polymer solutions as a function of the coating speed^51^. In a similar pattern, a high spinning speed for polymer-metal ion composite mixtures would increase the magnitude of centrifugal forces, enhancing thus the outward stretching and thinning of the precursor layer.

Lastly, the comparison of the microscopic morphology and thickness of the metal oxide layers prepared at 2000 RPM from two separate deposition mixtures, corresponding to samples A1/A2 (Fig. 3) and sample A (Fig. 4a-1,a-2), indicates a relatively poor batch to batch repeatability. The latter could be mainly associated with the transient state of the metal-organic precursor solution before and during spinning, due to the room temperature promoted alkoxide condensation.

### Resistive switching behavior of Cu/HfO_2_/p^++^ Si devices

**Figure 5:**
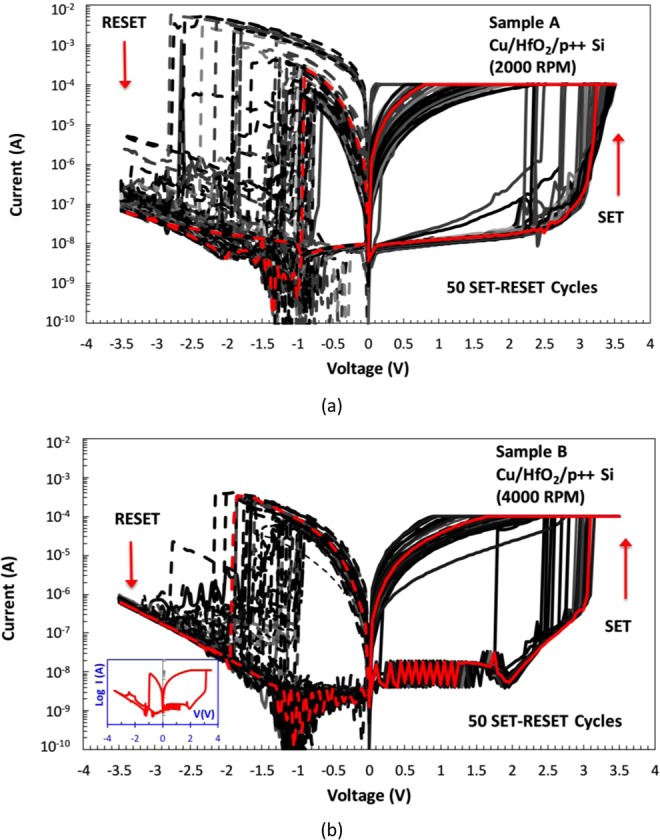
Semi-log plots of the *I-V* characteristic of 2 × 2 mm^2^ MR devices (Cu/HfO_2_/p^++^ Si) from **(a)** sample A and **(b)** sample B samples, showing 50 SET-RESET cycles. Si BE is grounded, while positive voltage is applied for the SET sweep, and negative voltage for the RESET sweep. The compliance current (CC) is fixed at 100 μA during the SET operation, while it is kept at 0.1 A during the RESET cycle. All of the 50 SET-RESET cycles are presented in grey scale, while one representative cycle is shown in red color to illustrate one single SET-RESET switching cycle. Arrows indicate the direction of the change in measured current during the SET and RESET transitions. Inset in (**b**) shows an example of single SET-RESET sweep cycle.

**Figure 6:**
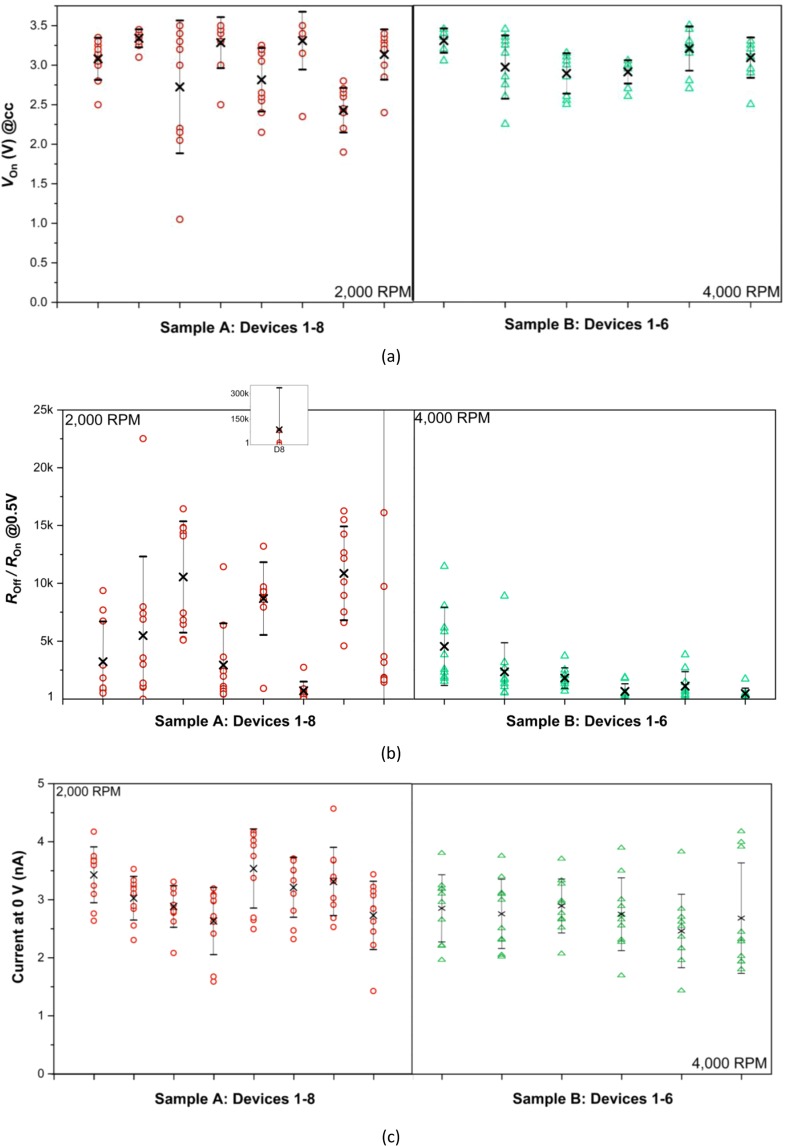
Summary of parameter values extracted from I–V curves for 10 SET-RESET cycles measured with different devices on samples A (red circles) and B (green triangles) **(a)** *V*_*on*_ (V), **(b)** *R*_*off*_/*R*_*on*_ Ratio and **(c)** Current at 0 V bias (nA). Also shown are the mean values per device (black ×).

The switching behavior of the (TE)-Cu/HfO_2_/p^++^ Si -(BE) stack was screened through the 6 to 8 randomly selected devices from samples A and B. The I-V characterization was carried out by grounding the p^++^ Si (BE) while sweeping voltage polarity at the Cu (TE) (see Fig. 2(a)). Figure 5(a,b) show the I-V curves for 50 consecutive SET-RESET cycles of a single device from samples A and B, respectively. The devices on both samples showed similar bipolar resistive switching, where memristors switch ON, transitioning from high resistance state (HRS) to low resistance state (LRS) in the positive polarity and then switch OFF (from LRS to HRS) in the negative polarity, as shown in Fig. 5(a,b). All of the tested devices on both samples did not require a preliminary forming step, which is a common characteristic for devices operating according to the electrochemical metallization mechanism (ECM), as established by Pan *et al*.^52^ and Menzel *et al*.^53^. The voltage value at which device switches ON from HRS to LRS, or in other words when OFF current, *I*_*off*_, increases and reaches the set compliance current (CC), is referred to as *V*_*on*_ and it ranges between 2.5–3.5 V for both samples (Fig. 6(a)). After this value, the device retains its low resistance state (remains ON) until it is switched OFF in negative polarity.

Figure 6 summarizes the measured values of *V*_*on*_ (at *I* = *I*_*CC*_), HRS and LRS resistance ratio (*R*_off_/*R*_on_) extracted at 0.5 V read voltage, and the capacitive current *I* evaluated at 0 V bias

According to Fig. 6(a) and Supporting Information (Fig. S5 results), some intra-device variations and large inter-device heterogeneity of the *V*_*on*_ parameter can be concluded for both memristor samples, A (population average = 3.0 V; RSD = 13%; N = 8; ANOVA *p*(*F* > *F*_critical_) = 4.8 × 10^−6^) and B (population average = 3.1 V; RSD = 8.1%; N = 6; ANOVA *p*(*F* > *F*_critical_) = 0) at 99% confidence. Although the average *V*_on_ parameter does not significantly depend on the spin-coating speeds examined and resulting oxide layer thicknesses, much of the fluctuations caused into this parameter can be associated with the thermal and interfacial behaviour of this layer as it develops during the fabrication process. With respect to the *R*_*off*_/*R*_*on*_ ratio parameter, the results of Fig. 6(b) show a population of single-device values ranging from 1 to 10^4^ for both samples (where an *R*_*off*_/*R*_*on*_ ratio value of 1 was observed occasionally when a device failed to reset). The overall *R*_off_/*R*_on_ population mean for sample B devices (2 × 10^3^; RSD 99%; N = 6) is approximately one order of magnitude smaller than that calculated for sample A devices (RSD 516%; N = 8). The intra-device variability for the *R*_off_/*R*_on_ values is relatively greater for sample A compared with sample B devices (see supporting Fig. S5), indicating a poorer electrical switching stability of the former stack. The enhanced intra-device cycling repeatability of sample B devices can be attributed to their denser, thinner, and hence more stabilizing conductive HfO_2_ layer (see Fig. 4). Nonetheless, at 99% confidence, the device-to-device heterogeneity of the *R*_off_/*R*_on_ parameter (see supporting Fig. S5) is greater across sample B memristors (N = 6; ANOVA *p*(*F* > *F*_critical_) = 4.5 × 10^−5^) as compared to sample A (N = 8; ANOVA *p*(*F* > *F*_critical_) = 0.31), and infers a substantial fabrication process dependency.

The overall magnitude of capacitive current is negligible (in the nA range) and does not significantly affect memristive properties of these devices, which can be concluded from zero-pinched hysteresis (Fig. 5).

**Figure 7:**
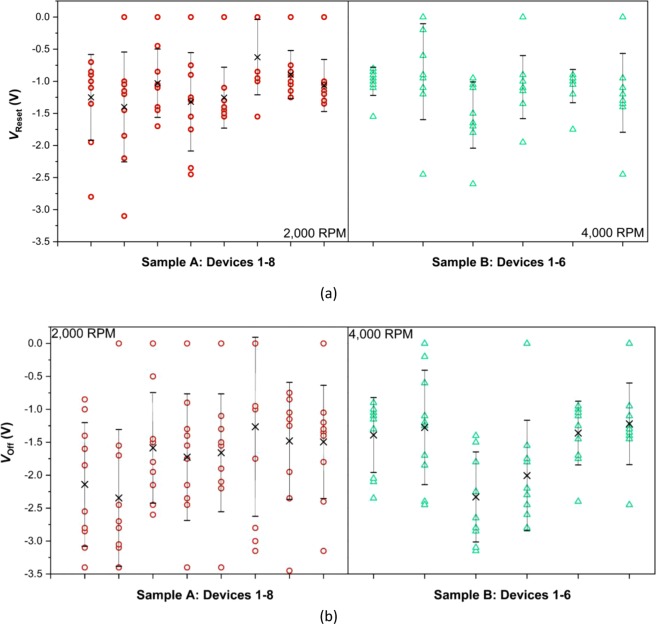
Summary of extracted measured **(a)** *V*_*reset*_ (V) and **(b)** *V*_*off*_ (V) values for 10 SET-RESET cycles for different devices on samples A (red circles) and sample B (green triangles). Also shown are the mean values for each device (black ×).

Figure 7 summarizes the *V*_*reset*_ and *V*_*off*_ values, extracted from 10 SET-RESET cycles for different devices on samples A and B. Large variations in the reset voltage can be observed for both samples and for all the tested memristors. Furthermore, the reset curves exhibit more than one switch-off event, with the initial drop in current occurring at the *V*_*reset*_^54^, while the device is fully switched off when LRS current (*I*_*on*_) becomes equal to HRS current (*I*_*off*_). For this work, a reset voltage (*V*_*off*_) is labelled as a voltage value when *I*_*on*_ = *I*_*off*_. With certain level of cycle-to-cycle variation, *V*_*reset*_ values are mostly between 0.5 and 1 V voltage (Fig. 7(a)), in agreement with most of the memristive devices reported in literature, as summarized by Ielmini *et al*. in^54^. Instances when *V*_*reset*_ is equal to 0 (Fig. 7(a)), indicate the cases when the device was already in a partially high resistive state at the start of the RESET process, which was more frequently observed with sample A devices.

For the rest of the analyses shown in this work, sample B devices were selected, due to their relatively suitable electrical properties but lower variations compared to sample A devices. In fact, a reduced cycle-to-cycle and device-to-device parameter variation is highly desired for sensing applications in order to reduce the detection uncertainty.

**Figure 8:**
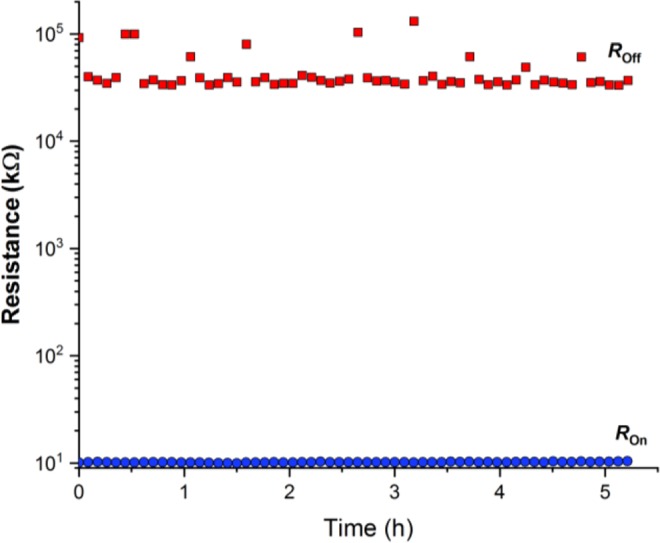
Retention test for a device from sample B with HRS and LRS resistance (*R*_*off*_ and *R*_*on*_) measured at 0.5 V reading voltage every 5 min over period of approximately 5 hours for each state.

Since the retention of resistive state is another important indicator of measurement reliability for sensing applications, the retention of a random device from sample B was tested over a period of five hours, during which both the HRS and LRS states were recorded. A five hours time window is a sufficiently long retention period for sensing applications as it provides the good level of confidence that the device will not change its resistive state during the given time period, except due to external factors (such as incoming radiation). The reading voltage is set equal to 0.5 V and the delay between two consecutive read pulses is kept at ~5 min. Figure 8 summarizes the results of a retention test, showing good retention and *R*_*off*_/*R*_*on*_ ratio above 10^3^. These results show that the sample B devices produced via the spin-coating methodology have good potential for use in sensing applications.

### Switching mechanism

The resistive switching can be divided into three main categories: (1) electrochemical metallization (ECM), (2) thermochemical mechanism (TCM) and (3) valence change mechanism (VCM). The Cu electrode is known to be an active electrode, leading to the formation of Cu ions by oxidative dissolution, diffusion of the latter species into the metal-oxide (acting as solid-state electrolyte), and re-conversion of those into conductive metallic paths, in agreement with ECM-based resistive switching^30,52,53,55,56^. On the other hand, VCM occurs due to field assisted oxygen anion migration^57^. While we can categorize conductive filament (CF) formation processes into these main three categories, identifying the governing pathway with asymmetric device structures can be challenging, especially when more than one mechanism coexist.

Resistive memories generally fail in practice to demonstrate the ideal behavior originally theorized by Leon Chua due to physical implications of the operating conditions onto the switching behavior^58^. Moreover, the system becomes more complicated and noncompliant with the ideal behavior when multiple switching mechanisms co-exist. Modeling of the device conduction mechanisms and extracting parameters such as ideality factor, density of states or oxide dielectric constants is usually performed individually for different regimes of operation. Some of the most commonly identified conduction mechanisms in resistive memories include: Schottky emission; Fowler-Nordheim tunneling (F-N); Direct tunneling; Poole-Frenkel emission (P-F); trap-assisted tunneling (TAT) and hopping conduction^57,59^.

To understand what is the dominant switching mechanism of devices presented in this work, a number of electrical characterization steps were carried out. For selected devices from sample B, four consecutive I-V sweeps were carried out with positive voltage applied to Cu electrode, ranging from 0 up to 1, 2 and 2.2 V (and back) without the occurrence of the switching event or transition to a lower resistive state (results shown in Supplementary File). Further, it was observed that during the DC bias tests, where different positive bias levels were applied to Cu electrode for a duration sufficient to ensure an equal flux for all consecutive measurements, the SET event was achieved when 2.5 V was applied at Cu electrode, while there was no evidence of stored charge in the device for voltage values of 2 V and less. These results imply that the resistive switching presented here (Fig. 5) is governed mainly by the conduction mechanisms that depend on the strength of the electric field.

**Figure 9:**
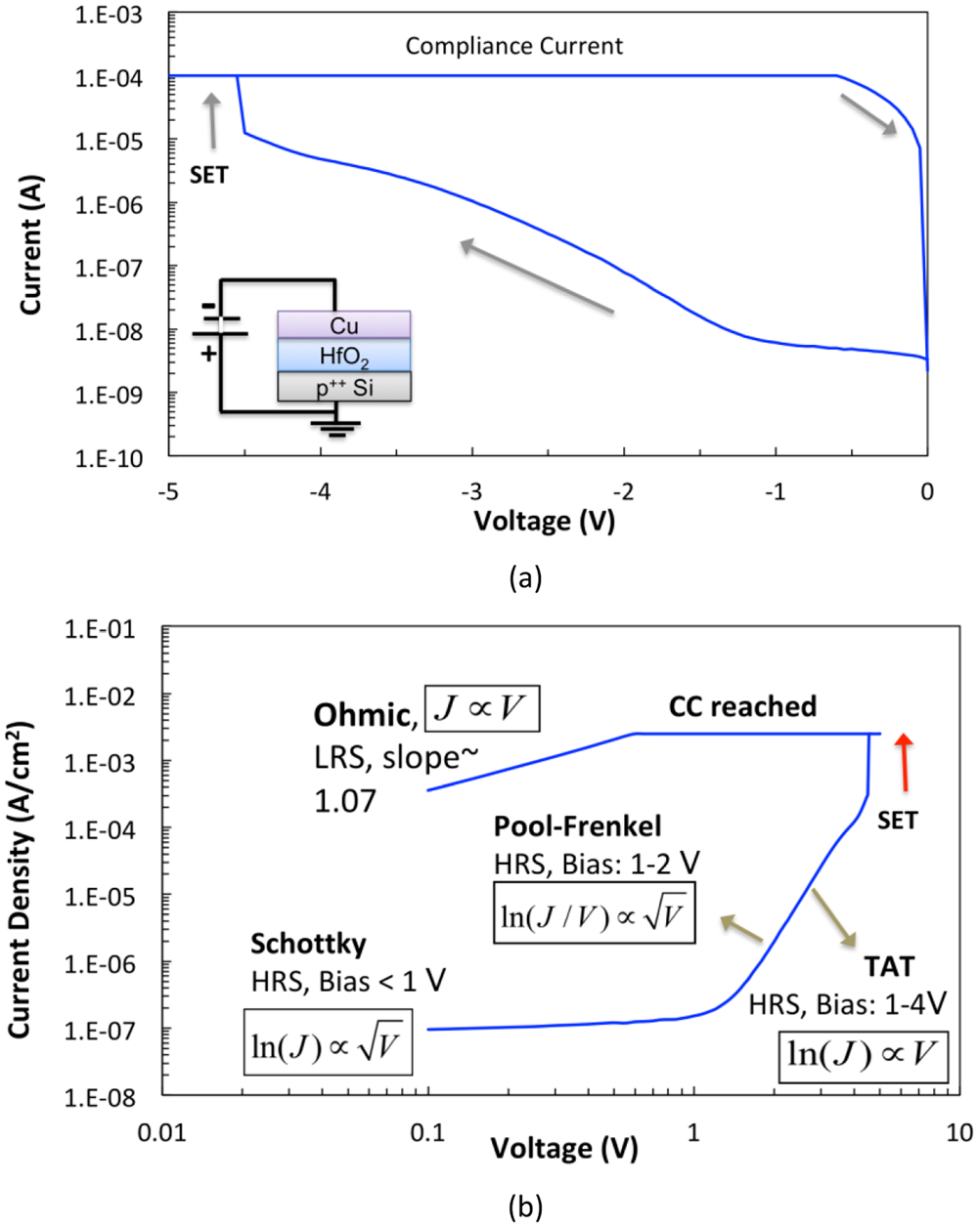
(**a**) I-V Characteristics of sample B, randomly selected, pristine device with TE Cu negatively biased and BE Si grounded. Results show switching due to non-ECM mechanisms for |V_on_| > 4 V and (**b**) Plot of log(*I*/*A*) vs. log *V* for negative bias, along with analysis of regions with different conduction mechanisms.

Moreover, sample B was tested with a negative bias applied to the Cu electrode of the pristine devices (device not tested before), in order to promote the oxygen vacancy based resistive switching and rule out any contribution of the Cu ions. The I-V characteristics for negative voltage sweep for sample B device are shown in Fig. 9(a), with the inset illustrating the testing configuration setup. Results show that resistive switching under negative bias occurs at bias value greater than 4.5 V, which is significantly larger than 3 V needed to achieve resistive switching under forward bias (Fig. 6(a)).

Furthermore, the I-V characteristics shown in Fig. 9(a) suggest that different conduction mechanisms are dominant at low and high bias values, for each LRS and HRS. Figure 9(b) summarizes the best fitting to main known conduction mechanisms for each region (details in Supplementary File and Fig. S4). For HRS, low bias (<1 V), data showed best consistency with Schottky emission model^60^. Further, for HRS, at bias 1–2 V, data had best fit to Pool-Frenkel emission^59^, implying that conduction is dominated by electrons hopping from trap states (oxygen vacancies) into conduction band under high electric field^57^. It can also be safely assumed that conduction in the high bias regime (1–4 V) is partly due to trap-assisted tunneling (TAT) via oxygen vacancies, as was confirmed by a number of studies of HfO_x_ based devices^57,60^.

Moreover, the effect of operating temperature on sample B devices is studied for switching under negative voltage bias (setup of the inset of Fig. 9(a)). The results are presented in the Supplementary File (Fig. S3) and suggest an oxygen-vacancy based conduction model, where an increased temperature could favor the formation of a greater number of oxygen vacancies due to enhanced oxygen diffusion^61^.

**Figure 10:**
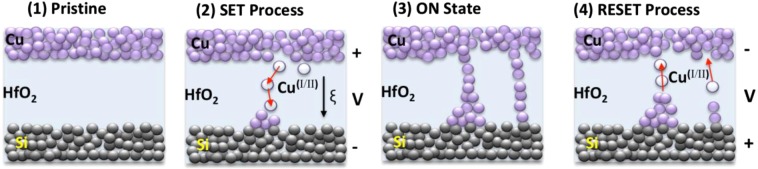
Schematic illustration of the prevalent electrical switching pathway, based on electrochemical metallization mechanism, occurring inside a (TE)-Cu/HfO_2_/p^++^ Si-(BE) memristor, showing multiple conductive filament paths, according to the experimental *I-V* characteristics presented in Fig. 5.

Additionally, the devices presented in this work do not require a pre-electroforming step, which is typical for ECM-based devices^53^. Haemori *et al*. have shown electroforming free resistive switching in Cu/HfO_2_/Pt devices, while similarly fabricated Pt/HfO_2_/Pt structure showed no hysteresis, confirming Cu role in this type of switching^30^. The ECM resistive switching in sample B devices can be explained by the electrochemical formation of Cu filaments, via the dissolution of positively biased Cu electrode into copper(I/II) cations and their migration along the applied electric field ξ, as illustrated in Fig. 10 (1)–(2)^52,53,55,62,63^. At the cathode side (grounded inert p^++^ Si electrode) the ions are reduced into Cu^0^ clusters at which the filamentary “growth” process of conductive metallic channels is initiated and continues towards the Cu electrode^52^, as shown in Fig. 10 (2). More than one filament can be formed during this process^52^, as was shown by Wu *et al*. using high-resolution transmission electron microscopy (HRTEM) scans of more than one conductive path in NiSi/HfO_2_/p^++^ Si transistors^64^. The conductive filament formation due to the reduction of Cu ions released from a positively biased Cu electrode was also confirmed by Lv *et al*. for Cu/HfO_2_/Pt RRAM devices using HRTEM scans after 500 switching cycles^65^, and by Choi *et al*. for Cu/GeTe layers^55^ and Qi Liu *et al*. for Cu/ZrO_2_ layers^63^ using *in-situ* cross-sectional TEM analyses.

From the results presented so far, we can speculate that Cu ion migration and Pool-Frenkel emission are the two main contributors to the resistive switching presented in Fig. 5 (when Cu TE is positively biased). However, ECM mechanism due to Cu migration can be considered as the dominant one, since the resistive switching occurs at the lower bias value (~3 V) vs. 4.5 V needed in the absence of Cu ions.

Furthermore, there is a relationship between the filament size and resistance in the LRS, as was explored by Gonzalez *et al*. for their Ni/HfO_2_/n^+^ Si RRAM devices^66^. The device reaches the ON state as soon as an electrical connection with the Cu electrode is established (Fig. 10 (3)), causing a sharp drop in the resistance value of the dielectric film. The reset process in bipolar ECM devices is driven by temperature and voltage induced migration of the ions, in addition to ion diffusion^52,54,62^. Once the bias polarity is reversed and compliance current removed (Fig. 10 (4)), the conductive filament is broken either by Joule heating (if sufficient bias is applied) or by reverse copper-ions migration^52^, which can take place via electromigration and diffusion^53^.

**Figure 11:**
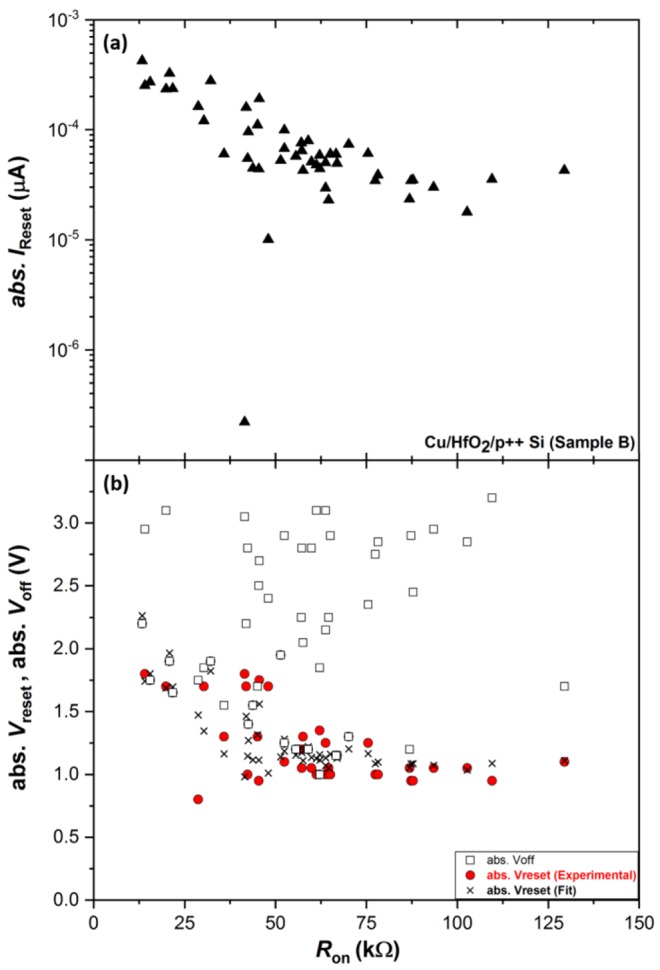
Measured *I*_*reset*_ (upper graph) and *V*_*reset*_ and *V*_*off*_ (lower graph, red circles and black squares, respectively) as a function of *R*_*on*_ extracted from I-V curves for 50 SET-RESET cycles for one device (sample B, Fig. 5(b)). Also shown are estimated (black ×) *V*_*reset*_ values using Eq. (1) where *R*_*s*_ ~ 3 kΩ, *V*_*reset*_′ ~ 0.981 with fit RMSE~0.318.

The universal model, proposed by Ielmini *et al*. in^54^, shows that the RESET current (*I*_*reset*_) and its corresponding voltage (*V*_*reset*_) are mostly dependent on the LRS resistance (*R*_*on*_), where device geometry and the metal oxide composition have little effect on *V*_*reset*_ and *I*_*reset*_ values. The values of *I*_*reset*_, *V*_*reset*_ and *V*_*off*_ were extracted from the I-V characteristics shown in Fig. 5(b) for 50 SET-RESET cycles of a single device from sample B. Figure 11 shows *I*_*reset*_, *V*_*reset*,_ and *V*_*off*_ as a function of the *R*_*on*_ values extracted at 0.5 V read voltage. The *I*_*reset*_ reduces with increasing *R*_*on*_ values in accordance with the universal memristor behavior reported by Ielmini *et al*.^54^.

*V*_*reset*_ and *V*_*off*_ (lower graph at Fig. 11) have two different trends as a function of *R*_*on*_. *V*_*reset*_ values are mostly around −1 V and are steadily decreasing with increasing *R*_*on*_, while *V*_*off*_ does not seem to depend on the *R*_*on*_. One explanation for *V*_*reset*_ decrease with increasing *R*_*on*_ is the effect of series resistance, as explained by Ielmini *et al*. in^54^. This hypothesis is confirmed here by implementing the relationship that relates the true *V*_*reset*_ (*V*_*reset*_′) with measured *V*_*reset*_, *I*_*reset*_ and estimated series resistance R_s_, shown in Eq. (1) ^54^.

1 $${V}_{reset}={{V}_{reset}}^{^{\prime} }+{R}_{s}{I}_{reset}$$

By iterative approximation to minimize the root mean square error (RMSE), the true *V*_*reset*_′ is estimated to be ~0.981 V with significantly high series resistance of *R*_*s*_~3 kΩ. The estimated *V*_*reset*_ values with an RMSE~0.318 are shown in the lower graph of Fig. 11 (black ×).

The difference between the *V*_*reset*_ and *V*_*off*_ values observed in our devices (Figs 7(a,b) and 11) can be explained by the presence of multi- or poly-filaments that can be simultaneously created in the oxide layer, as we have illustrated in Fig. 10 ^52,55,64^^,^. Wu *et al*. have shown in^64^ that spatially uncorrelated multiple conductive filaments can be formed inside the HfO_2_ layer during the SET process of Ni/Si/HfO_2_/p^++^ Si devices. Consequently, the RESET I-V data can exhibit multiple levels, where each filament is broken at a different voltage level, due to the presence and the variations in CF size and resistance values^64^.

### Application to gamma rays detection

Ionizing electromagnetic (EM) radiation detection and dosimetry, are subject of increasing interest in different fields, such as medical, military defense and space applications. EM radiation detectors are used either to facilitate human protection from accumulative and deleterious ionizing nature of the rays or to monitor and protect the endurance of electronic and other susceptible devices deployed industries and that are directly exposed to radiations^67,68^. A number of research has dealt with this problem^9,69–71^ with some exploring the capabilities of HfO_2_ for radiation detection and dosimetry in the configuration of various devices^72,73^, as well as using the memristive devices^74,75^.

**Figure 12:**
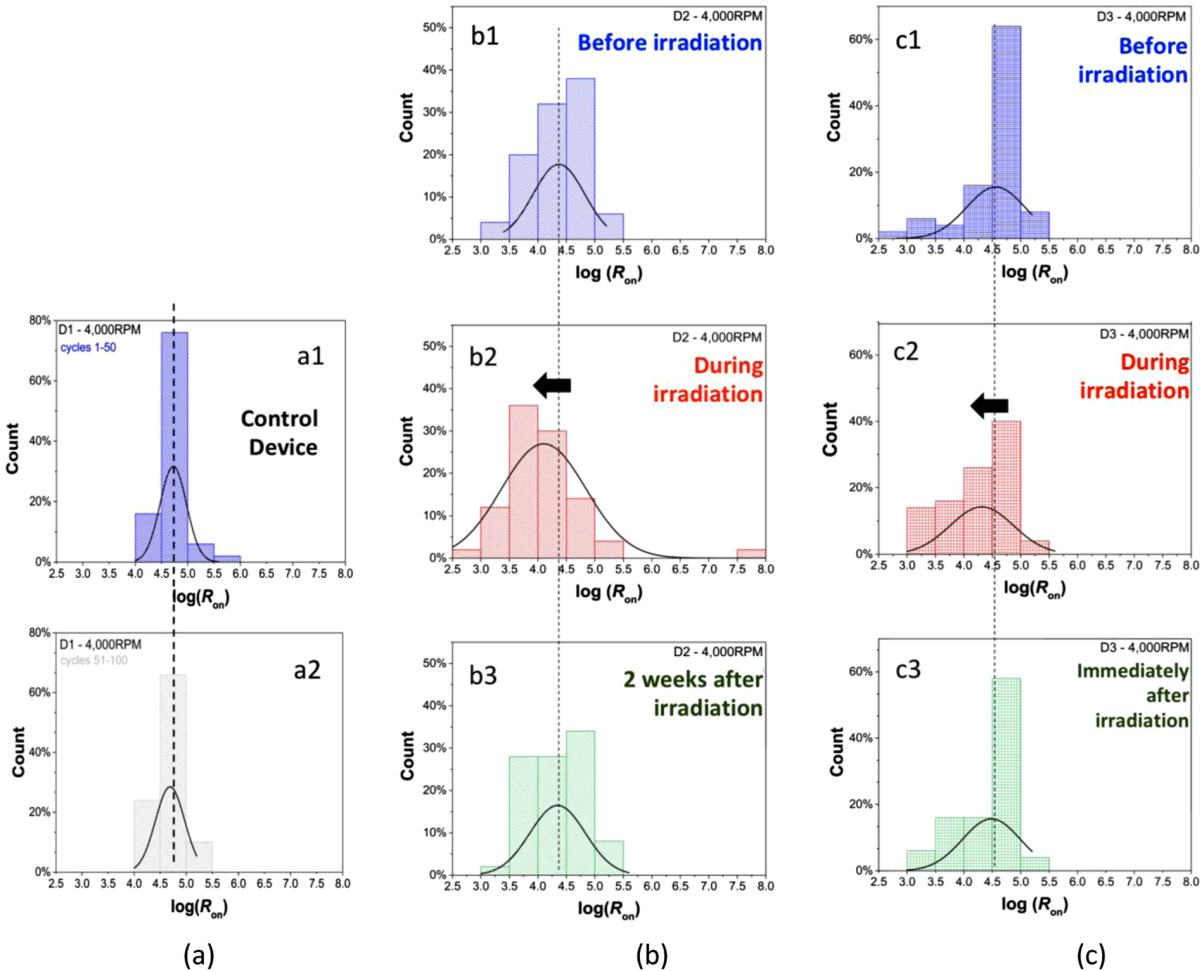
Probability distribution of log(*R*_*on*_) taken at 0.5 V reading voltage extracted from 50 SET-RESET cycles, each performed for **(a)** control device “D1” performed over 100 consecutive SET-RESET cycles, **(b)** device “D2” before, during and 2 weeks after irradiation and **(c)** device “D3” before, during and immediately after irradiation with γ–rays. Gamma rays are sourced simultaneously from four Cs-137 and two Am-241 point sources.

The sample B devices are tested under the exposure to the low activity γ-ray point sources, namely 662 keV Cs-137 and 60 keV Am-241, being positioned under the bottom electrode (p^++^ Si). Individual 2 × 2 mm^2^ memristors are cleaved away from the sample B in order to irradiate one device at a time. As a control, a device is tested over 100 SET-RESET cycles without radiation, in order to confirm that there is no significant statistical change in R_on_ values with an increasing number of sweep cycles (Fig. 12(a1,a2)). The other fresh devices are then tested for 50 SET-RESET cycles before, during and after exposure to gamma rays. Figure 12(b1,b3) show the probability distribution of log(*R*_*on*_) values extracted from measured data for device D1 before, during and two weeks after exposure to radiation, while Fig. 12(c1–c3) depict the results of a similar analysis for device D3 before, during and immediately after exposure to radiation. The data presented in Fig. 12(a1,a2,b1,c1) show that devices have mean *log(R*_*on*_) value approximately around 4.5, with standard deviations varying from sample to sample. The variability in the LRS resistance values (*R*_*on*_) can be attributed to the probabilistic nature of the number and strength of conducting filaments formed during the SET operation^76^.

Figure 12(b2,c2) show a clear shift towards lower value of log(*R*_*on*_) values for two tested devices, when exposed to gamma-rays. These results imply that the memristor’s conductive filaments are formed more strongly during the exposure to gamma-rays, increasing the probability of occurrences of lower *R*_*on*_ values. Finally, Fig. 12(b3,c3) show that after the radiation is removed, the devices statistical behavior appears to return to the initial state with slightly increased response variability immediately and two weeks after the exposure.

Further exploration of dominant radiation interaction mechanisms for devices presented in this work is required. However, it is likely that the mechanism of radiation registration by the memristor device occurs through ionic rather than charge production and collection that take place in CMOS devices and state-of-the-art radiation detectors^77–79^. Thus, radiation detection approach proposed in this paper has potential to reduce the power consumption of the detector, which could eliminate the need of significant amplification in the reading circuit required for memristor-based radiation detecting. This would further simplify the reading circuitry in memristor systems, which consequently helps achieving more compact device^12^.

## Discussion

This paper presented a novel and cost-effective sol-gel spin-coating method for the fabrication of memristive devices consisting of a (TE)-Cu/HfO_2_/p^++^ Si-(BE) stack. The oxide surface and memristive behavior were analyzed for different spin coating speeds. The devices exhibit a certain level of statistical variation in electrical parameters, such as *V*_*on*_, V_reset_, *V*_*off*,_ and *R*_*on*,_ mainly due to the materials and methods used in the device structure. Finally, devices are tested for their potential as radiation detectors. The results showed that this method produces devices with bipolar and ECM-dominant resistive switching, with statistical electrical repeatability over 100 consecutive sweep cycles and high *R*_*off*_/*R*_*on*_ ratio up to 10^4^. Stochastic nature of the *log(R*_*on*_) values for different devices is studied over 150 SET-RESET cycles, with 50 cycles carried before 50 during and 50 after the exposure to gamma rays. Results show that there is a statistical difference in *R*_*on*_ values before and during the gamma-ray exposure, where active radiation exposure enhances the building of conductive filaments and increases the probability of recording lower *R*_*on*_ values. The results presented here showcase a cost-effective, high yield memristor with potential for use in radiation sensing. The main advantage of memristor-based radiation sensing proposed here is the prospect of low power consumption needed to operate the detector compared to the state-of-the-art, in addition to simplified circuitry and possibility of more compact detecting device.

## Methods

### Device fabrication

The Cu/HfO_2_/p^++^ Si devices were fabricated using a sol-gel spin coating methodology to deposit the dielectric layer onto the p^++^ Si substrate. The deposition is followed with a post-thermal treatment to remove the organic residues, and finally the Cu TE are deposited by DC metal sputtering. Typically, an HfO_2_ precursor solution is prepared by vigorously mixing 0.15 g of hafnium isopropoxide isopropanol adduct (99% purity) in 0.25 g aqueous sulfuric acid (5 M), then mixing it with 0.25 g deionized (DI) water and 0.15 g formamide (99% purity). The aqueous sulfuric acid enables the dissolution of the precursor alkoxide by hydrolysis and complexation while the strongly acidic medium hinders the fast condensation. The formamide additive acts as a gelation control agent by releasing ammonia via gradual hydrolysis. The obtained mixture is first centrifuged at 3,000 RPM for 1 minute to separate the insoluble impurities. Then, approximately 0.77 g aliquot of the supernatant solution is gently mixed for 2 minutes with 0.40 g aqueous PVP (14% wt.). The composite organic-inorganic solution is inspected for air-bubble removal prior to spin coating, then deposited on 4 cm × 4 cm pre-cut p^++^ Si substrate pieces. The time used for the preparation of the precursor solution until its spin coating at room temperature is kept short (<15 min), to minimize the formation of macroscopic metal oxide lumps by condensation-induced aging. In this work, two different samples, A and B are spun-coated with the polymer-alkoxide composite solution for 30 s at 2000 and 4000 RPM, respectively. During spin coating, a small part of the p^++^ Si bottom electrode layer is masked with the tape in order to prevent its full coverage with the precursor layer and ensure easy access of the electrical probes to the surface for the electrical characterization studies (refer to Fig. 2). The spun-coated samples are dried on a hot-plate for 2 minutes at 90 °C, and then heat-treated at 500 °C for 2 hours (with a ramp-up temperature Δ*T* of 5 °C/min) to remove the residual solvent and organic content from the oxide layer. The Cu TEs are sputtered using Q300T T coating tool (Quorum Technologies, UK)^80^. During sputtering, a shadow mask is used for the deposition, where each Cu TE is designed as ~2 mm × 2 mm in size, and defines the area of one memristive device, as shown in Fig. 2a,b).

### Device characterization

The scanning electron microscopy (SEM) imaging was carried out to observe the spun-coated film morphology and estimate the film thickness for different devices. The sample cross-sections were obtained by dicing the wafer pieces into smaller samples using a diamond-tip scriber, and observing those with SEM without applying a conductive coating. Imaging was performed with a JSM-7610F Schottky field-emission electron microscope (JEOL LTD., Japan) and Nova NanoSEM 650 (FEI Company, USA) using a secondary electron detector, a working distance range of 5 to 10 mm, and an acceleration voltage of 5 or 10 kV. A Keithley 4200-SCS Parameter Analyzer (Tektronix, USA) was used for the electrical characterization of the fabricated devices. The current-voltage (I-V) sweeps were applied for the measurements of the SET and RESET operations, where the bottom Si electrode was kept grounded. A positive voltage sweep from 0 to 3.5 V was applied on the TE (Cu) for the SET operation, and a negative one from 0 to −3.5 V was supplied during the RESET. All tested devices from samples A and B were characterized through the same sweeping protocol, using a voltage step size of 0.05 V. During the SET sweeps, a compliance current *I*_*CC*_ = 100 μA was maintained. During the RESET process a tool compliance of 0.1 A, was applied in order to allow a sufficient amount of Joule’s heating to switch OFF the device.

### Statistical data analysis

Origin Pro 2015 software was used for graphing and statistical data treatment. The analysis of variance and comparison of means across multiple devices was carried out with the one-way ANOVA test based on 10 randomly pooled SET-RESET data. The device-to-device electrical performance variations within sample A and sample B structures were assessed according to the Levene’s test, at 99% confidence. The means comparison plots were generated according to the Scheffe’s test (see Supplementary Figs S5 and S6). For the radiation studies, the log(*R*_on_) values from 50 consecutive SET-RESET cycles were found to be normally distributed at 99% confidence, where the goodness of the normality fitting for the probability distributions was verified with the Kolmogorov-Smirnov (K-S) nonparametric test. Accordingly, the comparison of the effects of radiation exposure on the electrical properties of the examined devices was established by plotting the probability distributions of the log(*R*_on_) values according to the Gaussian model. Moreover, the comparison of means for the log(*R*_on_) values obtained before, during and after exposure to radiation was fulfilled with Fisher Least Significant Difference (LSD) test at 95% confidence, via the one-way ANOVA (see Supplementary Figs S7 and S8).

## Supplementary information

Supplementary information
