# Supplementary material for: Bipolar Cu/HfO2/p++ Si Memristors by Sol-Gel Spin Coating Method and Their Application to Environmental Sensing
Source: Sci Rep. 2019 Jul 10;9:9983. doi: 10.1038/s41598-019-46443-x (PMC6620357; doi:10.1038/s41598-019-46443-x)
Supplement: Supplementary file 1 — Supplementary information [file 41598_2019_46443_MOESM1_ESM.doc]

Bipolar Cu/HfO_2_/p^++^ Si Memristors by Sol-Gel Spin Coating Method and Their Application to Environmental Sensing

**Sabina Abdul Hadi^1,2^, Khaled M. Humood^1^, Maguy Abi Jaoude^3^, Heba Abunahla^1^, Hamda Faisal Al Shehhi^4^, and Baker Mohammad^1*^**

^1^Department of Electrical and Computer Engineering*,* Khalifa University of Science and Technology, P.O. Box 127788, Abu Dhabi, UAE

^2^Currently working at College of Engineering and IT, University of Dubai, P.O. Box 14143, Dubai, UAE

^3^Department of Chemistry, Khalifa University of Science and Technology, P.O. Box 127788, Abu Dhabi, UAE *^4^*UAE Space Agency, 7133, Abu Dhabi, UAE

*Correspondence to: [baker.mohammad@ku.ac.ae]

# Supplementary Information

**Switching Mechanism** The devices governed by VCM switching mechanisms change their resistive states with increasing flux that the devices are exposed to, while devices governed by ECM mechanism strongly depend on the field strength (applied bias).

In order to observe the dominant switching mechanisms for the devices presented in this work, we tested sample B devices in two main configurations:

(i) “Configuration 1” when the Cu (TE) electrode is positively biased and Si is grounded, and

(ii) “Configuration 2” where the Cu (TE) is negatively biased and Si is grounded, making Si effectively positively biased during the voltage sweep.

In the “Configuration 1”, Cu ions are known to contribute to resistive switching (ECM), while in “Configuration 2” we expect absence of Cu ions and resistive switching to be due to different factors, such as oxygen vacancies, charge trapping, tunnelling.

**Configuration 1: (+V) - Cu/HfO_2_/p^+^ Si – (0 V)**

In the first configuration, the dissolution and formation of metallic Cu filaments is likely to contribute to the resistive switching via ECM.

Fig. S1 shows I-V characteristics for 10 consecutive positive sweeps for a sample B device, all performed without a negative (reset) sweep in between (Cu electrode is positively biased, while Si is grounded). In Fig. S1(a), voltage sweeps with final voltage values varying between 1 to 2V are shown while no resistance change is observed in the device. Further, the voltage sweep is increased up to 2.2V and repeated three times (Fig. S1 (b)) in order to see if exposing the device to positive bias repeatedly will permanently change its resistive state by movement of oxygen vacancies. However, device retains its initial high resistive state throughout these 6 consecutive positive voltage sweeps. Finally, the voltage bias is increased to 2.5 V where device changes its resistive state (Fig. S1 (c), 7^th^, blue curve). Moreover, the device is exposed to two more positive voltage sweeps up to 2.5V, but its resistance change does not increase beyond the initial state achieved with the first sweep up to 2.5V (curves 7^th^-9^th^). Only after the final bias value is increased to 3 V, the device is able to reach set compliance current.

Similar behavior is observed for a device from sample A. These results suggest that switching in Cu/HfO_2_/p^++^Si devices, when Cu electrode is positively biased, is strongly dependent on the strength of the electric field rather than the flux affecting the device, in agreement with ECM based switching mechanism.


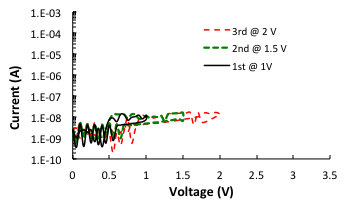

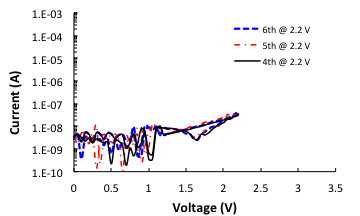


**(a) (b)**


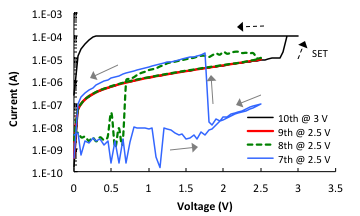


**(c)**

**Figure S1.** Semi-logarithmic plot of current-voltage characteristics for sample B device for **(a)** four consecutive positive sweeps where voltage bias is varied between 0 and 1-2.2 V, **(b)** three consecutive positive sweeps where voltage bias is varied between 0 and 2.2 V each time and **(c)** four consecutive positive sweeps where voltage bias is varied between 0 and 2.5- 3V. Total 10 consecutive positive sweeps are shown in (a)-(c) without reset sweep in between.

To further differentiate if VCM or ECM mechanism is dominating the switching behavior presented in this work, the effect of the electric flux on the resistive switching of the device is explored. Typically, a device is subjected to a fixed amount of electric flux (~100 Wb) at different instances, by applying different values of positive DC voltage bias to the Cu electrode (“Write” bias = 1V, 1.5V, 2 and 2.5V) for appropriate time durations to produce an equal amount of flux (
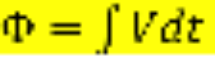
*≈*100 Wb). The value of the total built-up instantaneous charge during the “Write” procedure is extracted from the measured DC current-time characteristics (
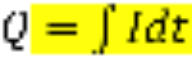
) and plotted in Fig. S2 (a) for two sets of experiments: one where a RESET sweep is applied in between each DC bias test (green) and another where consecutive DC bias measurements are carried out without any RESET operation. After each “Write” process corresponding with the application of 100 Wb, the stored charge is “read” at 0.5V for a brief time and its value is plotted in Fig. S2 (b). Results in Fig. S2 (a) show that for bias values up to 2 V, approximately equal amount of charge is built-up when 100 Wb is applied, while no evidence of increase in the stored charge is observed after the “Read” process (Fig. S2 (b)). Once 2.5 V is applied, the device switches ON even before the flux amount of 100 Wb is reached, resulting in an increase of built-up, as well as stored charge. For VCM dominated devices, it is expected that the consecutive application of flux, without the subsequent reset, would result in an increasing amount of stored charge (i.e. gradual change in resistive state), but that is not the case for the devices presented in this work. The analysis shown in Fig. S2 is for the device from sample B, but similar findings were observed for sample A devices. These results confirm that the resistive switching observed with spun-coated Cu/HfO_2_/p^++^Si devices when the Cu electrode is positively biased, is not predominantly flux based, but rather field driven, which is a characteristic of the ECM switching mechanism.

#

**Figure S2.** (a) Built–up charge during applied flux of ~ 100 Wb with (green) and without (red) RESET sweeps in between and (b) Stored charge measured over period of 2 s after “Write” bias equivalent to flux of 100Wb due to different bias values.

**Configuration 2: (-V)- Cu/HfO_2_/p+ Si – (0 V)**

In “Configuration 2” where Cu (TE) is negatively biased during a SET operation, we expect that a resistive switching is generated by different factors, such as oxygen vacancies migration, charge trapping and/or tunnelling, as opposed to the dissolution/formation of conductive Cu filaments from the Cu electrode.

In this configuration, the resistive switching of a fresh device is tested when a negative bias is applied to the Cu (TE), while the Si bottom electrode is grounded, so that no ECM switching event is induced. The Figure 9 in the main manuscript shows the I-V characteristics of a “pristine” sample B device, tested at 25 ºC, using “Configuration 2”. The results indicate that when the Cu (TE) is connected to a negative voltage supply, the resistive switching occurs at a bias magnitude greater than 4 V (in the negative polarity mode). This threshold switching condition is beyond the experimental voltage range examined in the present work (-3.5 🡨 0 and 0 🡪 +3.5), which further suggests that the contribution this secondary switching toward the established I-V curves is minimal.

To further confirm the presence of vacancy based conduction mechanism, another random fresh device from sample B was tested with consecutive sweeps of negative voltage values of up to -3.5V, applied at the Cu electrode by gradually increasing the operating temperature from 25, to 50 then 75 °C. Accordingly, three consecutive sweeps were performed at each temperature. The temperature dependent I-V characteristics for the device tested under “Configuration 2” are shown in Figure S3, where for the temperatures below 50°C device does not switch at bias values up to -3.5 V. However, when the operating temperature is increased to 75 °C, the device switched on sharply and remained in an ON state. This observation suggest an oxygen-vacancy based conduction model, where an increased temperature could favor the formation of a greater number of oxygen vacancies due to enhanced oxygen diffusion^61^.


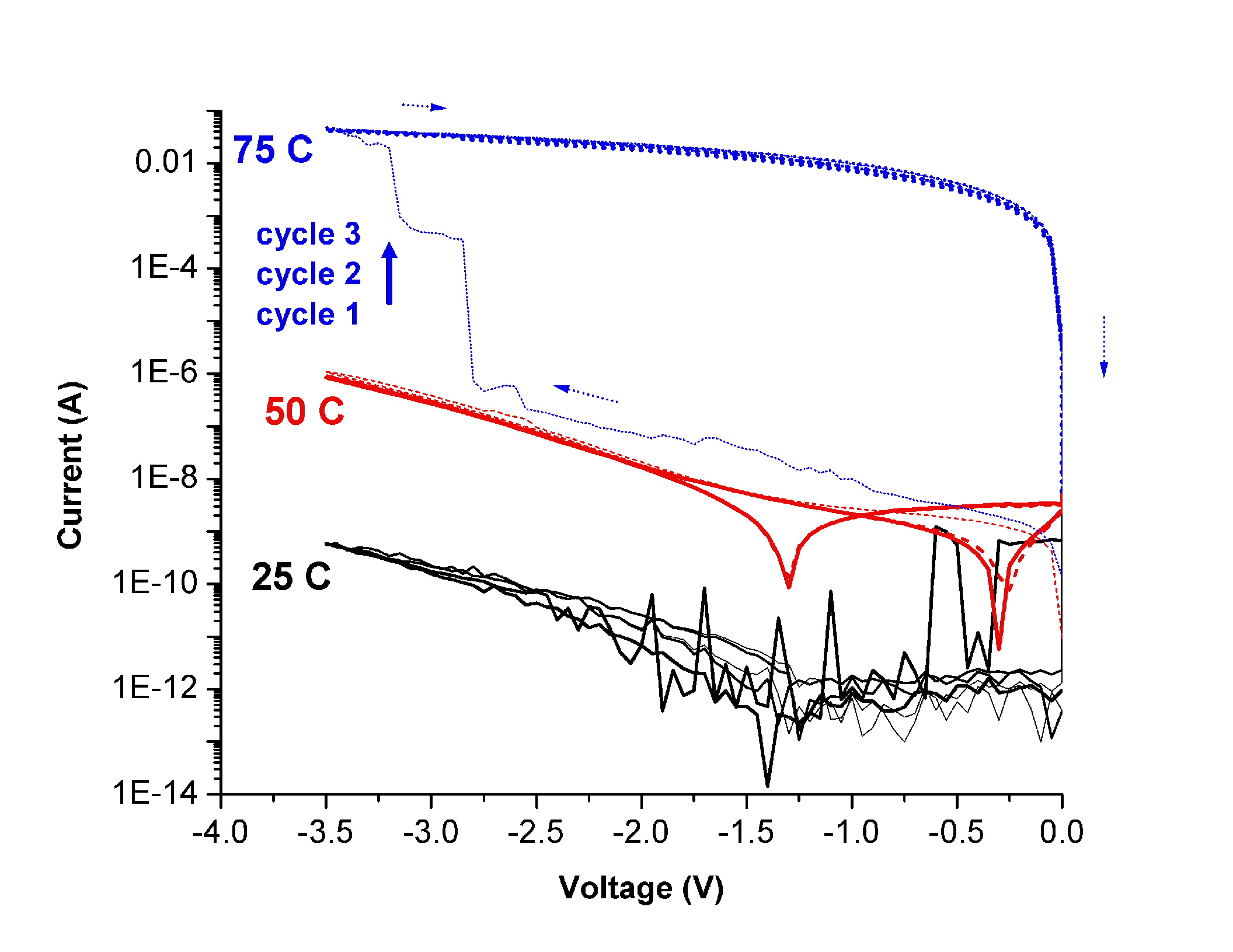


**Figure S3.** I-V Characteristics of Cu/HfO_2_/p++Si sample B device for consecutive voltage sweeps measured at three different operating temperatures (25, 50 and 75 °C). Three consecutive sweeps are performed at each temperature value, before increasing the temperature to the next level.

**Conduction Mechanism Analysis**

To identify individual conduction mechanisms, I-V curve shown in Fig. 9 (a) of the main text is analysed for the best fit to known conduction mechanisms. When current density, J, is directly proportional to voltage bias, V, an Ohmic relationship can be deduced (as shown in Fig. 9(a)). Furthermore, for regions when current ln(J/V) is proportional to
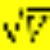
 a Pool-Frenkel^59^ conduction can be assumed (Fig. S4 (a), while for regions when
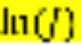
 is proportional to
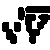
 and
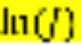
 is proportional to V, a Schottky emission^60^ and trap-assisted tunnelling ^57, 60^ can be assumed, respectively (Fig. S4 (b) and (c).


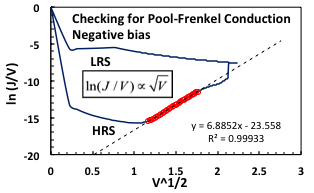

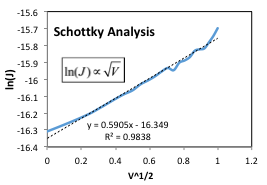

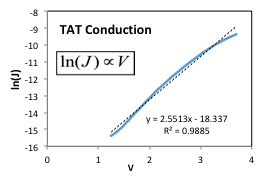


Figure S4. Details of IV-data analysis for the best fit to known conduction mechanisms (a) Pool-Frenkel, (b) Schottky emission and (c) Trap-assisted tunnelling fitting.

**Statistical Data Analysis**

(i) Electrical Performance Measurements – device-to-device variations for samples A and B.


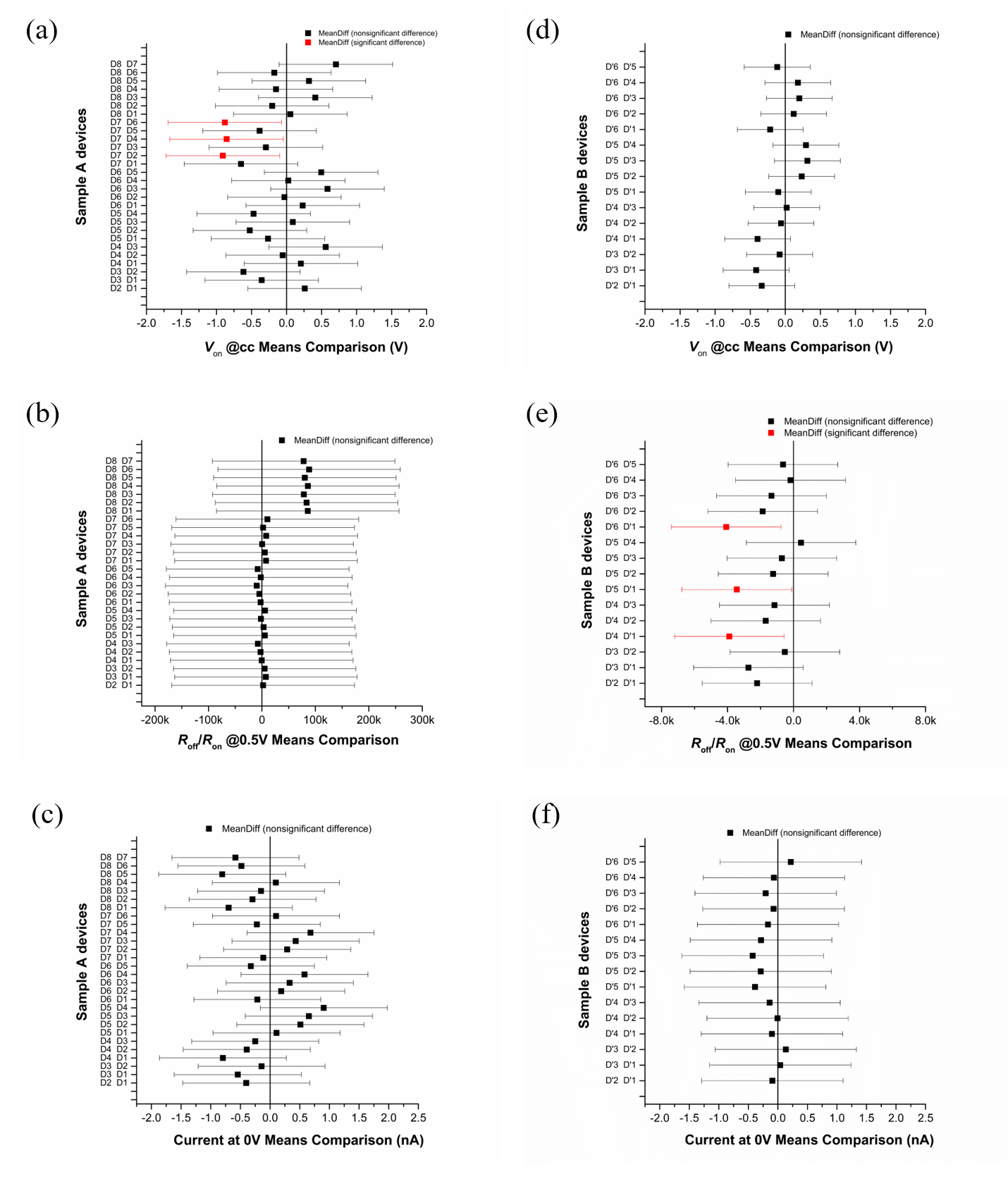


**Figure S5.** Means comparison plots of the electrical performance parameter measurements (V_on_, R_off_/R_on_, and current at 0 V) for 8 sample A devices (a-c) and 6 sample B devices (d-f). The data were pooled from 50 random SET-RESET cycles.


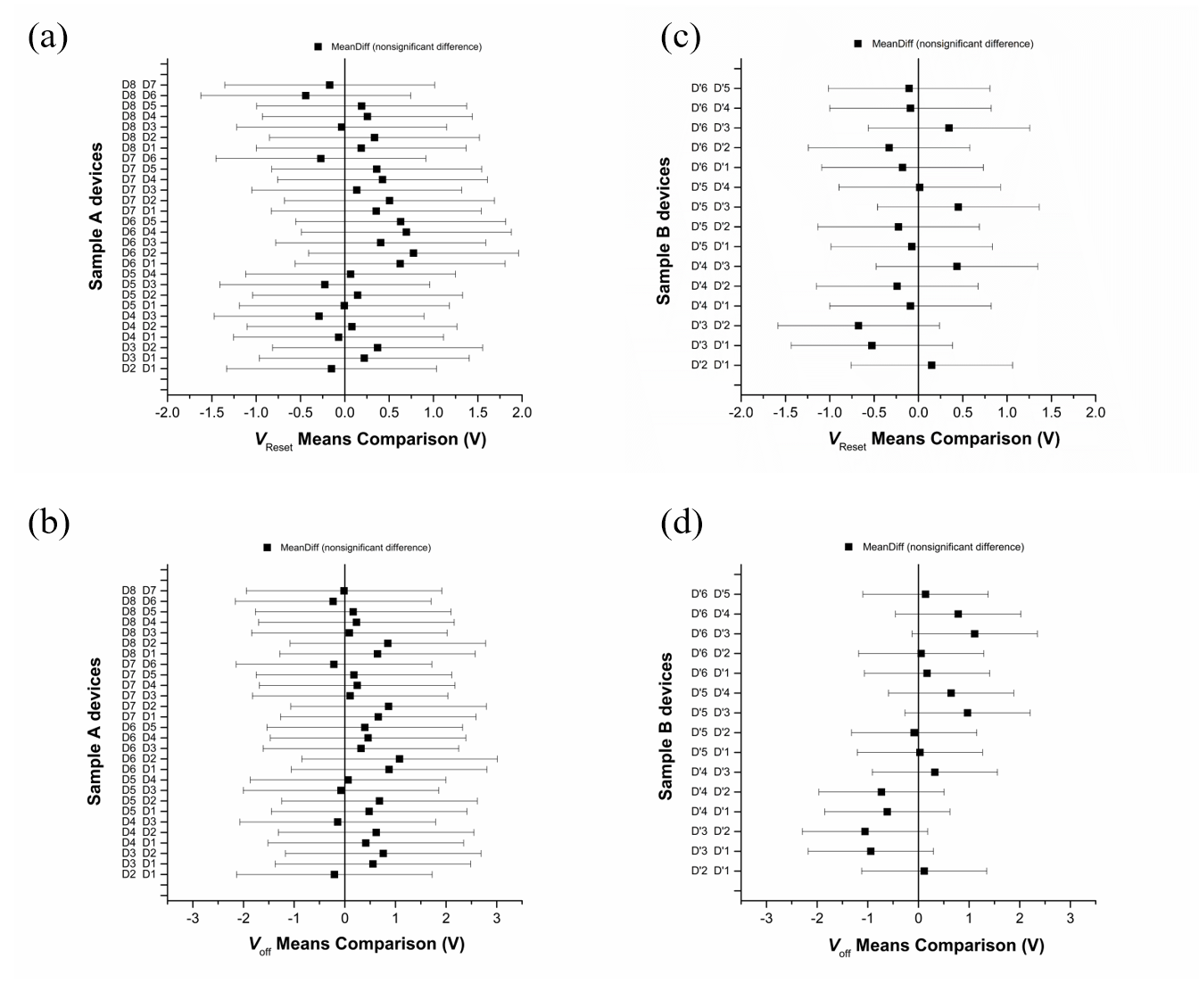


**Figure S6.** Means comparison plots of the electrical performance parameter measurements (V_off_ and V_reset_) for sample A devices (a,b) and sample B devices (c,d). The data were pooled from 50 random SET-RESET cycles. The plots were produced using a one-way ANOVA with the Scheffe’s test at 99% confidence.

(ii) Radiation studies – single device log(Ron) performance and device-to-device variation, before, during and after exposure to soft gamma ray sources.


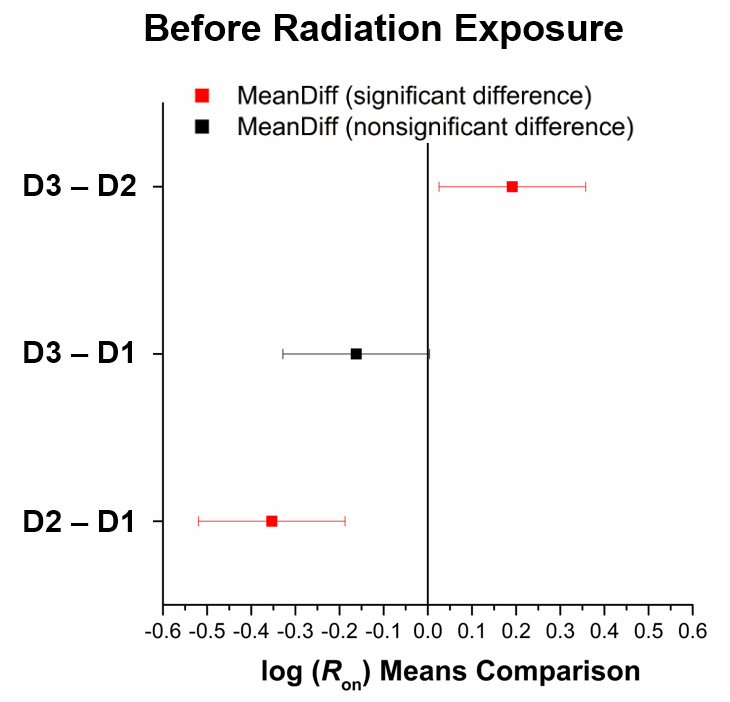


**Figure S7.** Means comparison plot of the log(Ron) values for three sample B devices. The data were obtained from 50 consecutive I-V sweep cycles recorded before irradiation. The plots were produced using a one-way ANOVA with the Fisher’s Least Significant Difference (LSD) test at 95% confidence. At this level, the statistically meaningful differences were observed on the log(Ron) confirm some device-to-device parameter variability.


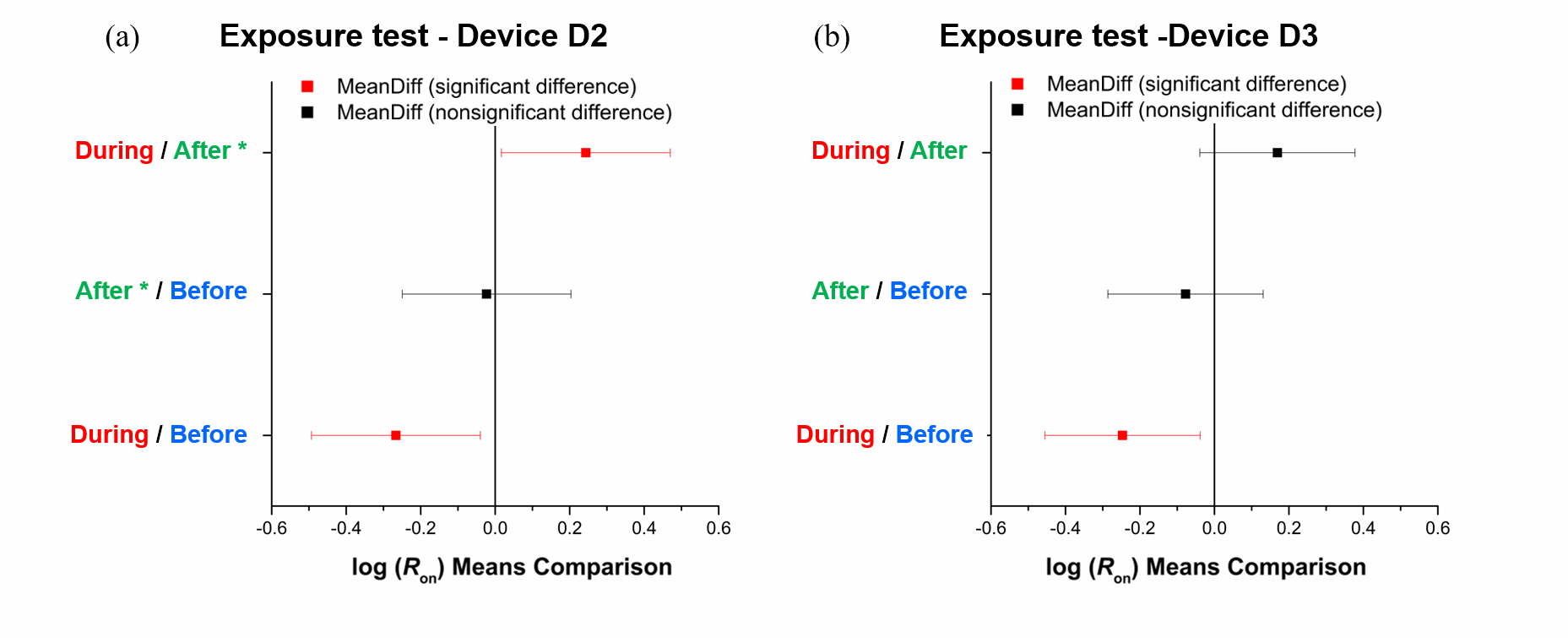


**Figure S8.** Means comparison plots of the log(Ron) values for devices (a) D2 and (b) D3 shown in Figure S6. The data were obtained from 50 consecutive I-V sweep cycles recorded before, during and after exposing the device to Cs-137 (662 keV) and Am-241 (60 keV) radioactive gamma ray sources. The plots were produced using a one-way ANOVA with the Fisher’s Least Significant Difference (LSD) test at 95% confidence. At this level, statistically meaningful differences were observed on the log(R_on_) values when both devices were irradiated, regardless of their initial log(R_on_) values.
